# Genetic diversity of wild rice accessions (*Oryza rufipogon* Griff.) in Guangdong and Hainan Provinces, China, and construction of a wild rice core collection

**DOI:** 10.3389/fpls.2022.999454

**Published:** 2022-10-03

**Authors:** Jing Zhang, Dajian Pan, Zhilan Fan, Hang Yu, Liqun Jiang, Shuwei Lv, Bingrui Sun, Wenfeng Chen, Xingxue Mao, Qing Liu, Chen Li

**Affiliations:** Rice Research Institute, Guangdong Academy of Agricultural Sciences, Guangdong Provincial Key Laboratory of New Technology in Rice Breeding, Guangdong Rice Engineering Laboratory, Guangzhou, China

**Keywords:** genetic diversity, SNP, *O. rufipogon*, core collection, multiplex PCR (mPCR)

## Abstract

*Oryza rufipogon* Griff. is a valuable germplasm resource for rice genetic improvement. However, natural habitat loss has led to the erosion of the genetic diversity of wild rice populations. Genetic diversity analysis of *O. rufipogon* accessions and development of the core collection are crucial for conserving natural genetic diversity and providing novel traits for rice breeding. In the present study, we developed 1,592 SNPs by multiplex PCR and next-generation sequencing (NGS) technology and used them to genotype 998 *O. rufipogon* accessions from 14 agroclimatic zones in Guangdong and Hainan Provinces, China. These SNPs were mapped onto 12 chromosomes, and the average MAF value was 0.128 with a minimum of 0.01 and a maximum of 0.499. The *O. rufipogon* accessions were classified into ten groups. The mean Nei’s diversity index and Shannon–Wiener index (I) were 0.187 and 0.308, respectively, in all populations, indicating that *O. rufipogon* accessions had rich genetic diversity. There were also differences in the genetic diversity of *O. rufipogon* resources in the 14 regions. Hainan populations possessed higher levels of genetic diversity, whereas the Guangzhou population had lower levels of genetic diversity than did the other populations. Phylogenetic analysis revealed that the genetic relationship among the distribution sites of *O. rufipogon* was closely related to geographical location. Based on genetic distance, a core collection of 299 accessions captured more than 99% of the genetic variation in the germplasm. This study provides insights into *O. rufipogon* conservation, and the constructed core collection provides valuable resources for future research and genomics-assisted breeding of rice.

## Introduction

In recent times, with the popularization of single varieties, pressure from environmental selection and the aggravation of pest and disease issues, the genetic basis of cultivated rice has inevitably narrowed (Swain et al., 2017). It is urgent that rice breeders enrich the genetic diversity of cultivated varieties and breed new varieties with better rice quality, higher yield and stronger resistance (Atwell et al., 2014). Crop wild relatives (CWRs) retain genetic diversity and thus represent a valuable genetic resource for modern agriculture (Zhang et al., 2017).

*O. rufipogon* Griff. (2n = 24, AA), an important CWR of cultivated rice, has accumulated many important agronomic characteristics over its long evolution, such as wide adaptability, resistance to insects, diseases and abiotic stresses, cytoplasmic male sterility, and better quality, which are desirable for the improvement of rice crops (Song et al., 2005; Henry, 2022). As one of the origins of cultivated rice in Asia, China has abundant wild rice resources, which are distributed in Guangdong, Guangxi, Hainan, Yunnan, Hunan, Jiangxi, Fujian and Taiwan (Liu et al., 2016). Guangdong and Hainan Provinces are in southern China and have favourable light and heat conditions. In this area, wild rice is widely distributed and exhibits abundant morphological and genetic diversity. Moreover, researchers have shown that South China is a centre of genetic diversity for wild rice (Li et al., 2006; Song et al., 2005). Therefore, *O. rufipogon* of Guangdong and Hainan Provinces play an important role in rice breeding improvement and basic research in China. However, the natural habitats of wild rice are rapidly shrinking due to urban and industrial occupation, and the large quantity of *O. rufipogon* resources also makes research and utilization difficult (Vaughan et al., 2008; Lakew et al., 2021). Hence, it is essential to identify the genetic diversity and develop efficient methods to conserve and make use of these natural *O. ruﬁpogon* populations.

Molecular marker analysis is one of the most useful methods of investigating the genetic diversity of *O. rufipogon* because of its rapid, simple, environmentally friendly and reliable detection results (Sarif et al., 2020). In wild rice, DNA markers such as inter-simple sequence repeat (ISSR) (Qian et al., 2001; Girma et al., 2010) and microsatellite or simple sequence repeat (SSR) (Zhou et al., 2003; Melaku et al., 2013) markers are increasingly being used to identify and evaluate genetic variation. In addition, advanced NGS technology has enabled timely mining of high-density SNP information throughout the genome for genetic identification at a significantly reduced cost (Singh et al., 2016; Xu et al., 2016; Ab Razak et al., 2020).

Core collection (CC) construction is a favoured approach to the efficient exploration and conservation of novel variation in genetic resources (Zhang et al., 2011). A core germplasm is a fraction of resources representing the maximum genetic diversity of the total population (Liu et al., 2020). The research showed that the proportion of core germplasm was different for different crops, generally varying from 5 to 30%. At present, the core germplasm of cultivated rice has been established in most parts of the world (Abadie et al., 2005; Yan et al., 2007; Zhang et al., 2011; Kumar et al., 2020; Tanaka et al., 2020). In wild rice, the optimal primary core collection of *O. rufipogon* was acquired using 5571 accessions from the national genebank based on agronomic and morphological characters (Yu et al., 2003). Pan et al. (2018) explored the genetic diversity and population structure of 4173 *O. rufipogon* in Guangxi, China, and developed a core collection.

There are abundant *O. rufipogon* resources in Guangdong and Hainan Provinces, China. However, previous research was limited to individual populations from a few ecological regions. The genetic diversity of *O. rufipogon* in Gaozhou, Guangdong, China, was studied (Chen et al., 2009). A total of 110 *O. rufipogon* accessions from four subregions (Suixi, Enping, Lufeng, Fogang) in Guangdong Province was studied for preserving the genetic diversity (Zhang et al., 2021). In this study, 998 elite *O. rufipogon* accessions were selected from almost *O. rufipogon* distribution areas of Guangdong and Hainan Provinces and genotyped using multiplex PCR and NGS methods. Analyses were carried out as follows. First, the genetic diversity of the accessions was assessed. Then, a core collection of common wild germplasms was constructed. Finally, we evaluated the diversity of core germplasm resources.

The demonstration of genetic diversity and establishment of a CC would provide a scientific basis for the classification, conservation, and innovative utilization of these precious wild rice resources.

## Materials and Methods

### Plant materials

**Figure 1:**
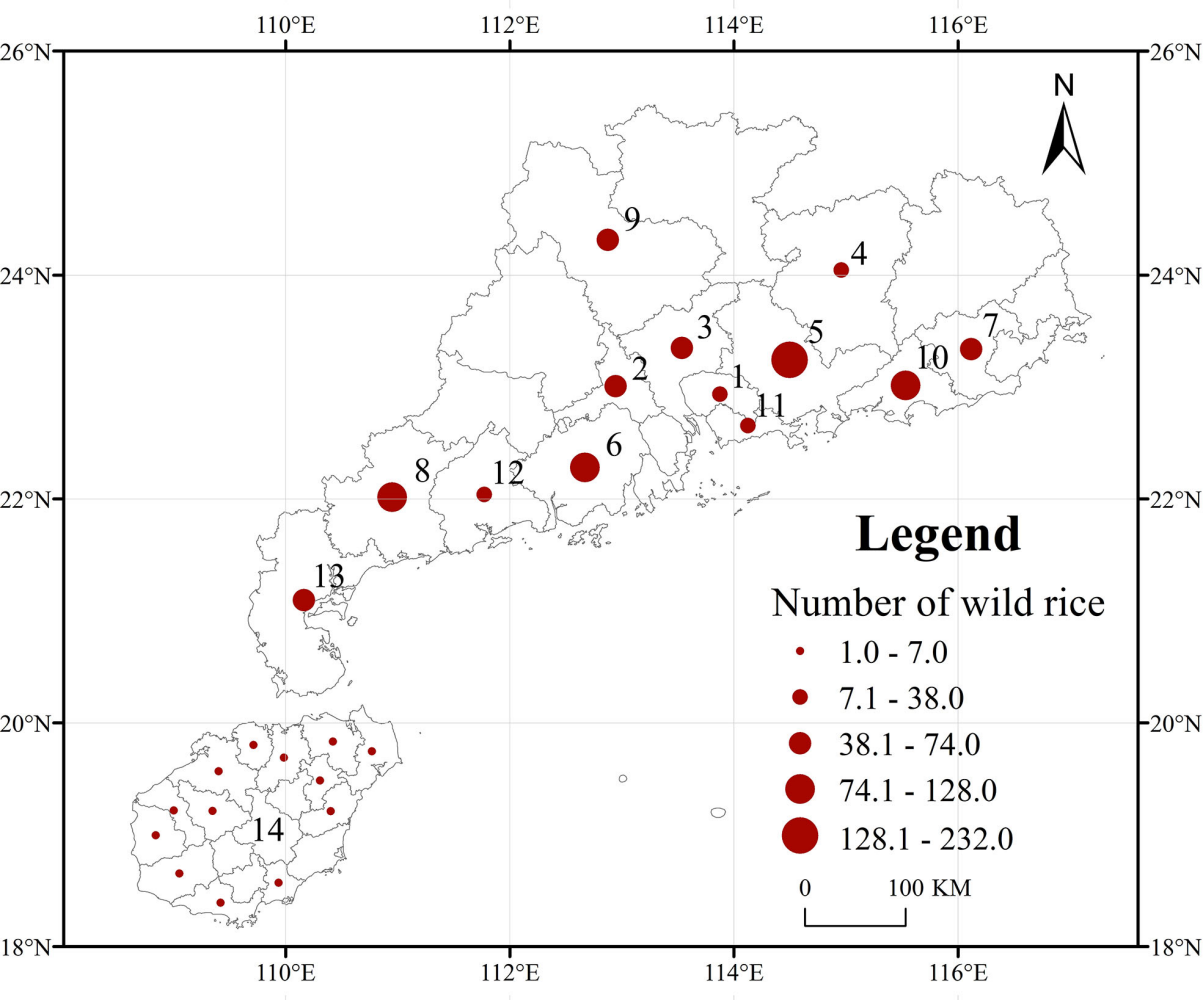
Ecogeographical distribution of 998 representative *O. rufipogon* accessions in Guangdong and Hainan Provinces, China. The circle size indicates the relative sampling number from each region. 1: Dongguan, 2: Foshan, 3: Guangzhou, 4: Heyuan, 5: Huizhou, 6: Jiangmen, 7: Jieyang, 8: Maoming, 9: Qingyuan, 10: Shanwei, 11: Shenzhen, 12: Yangjiang, 13: Zhanjiang; 14: Hainai.

A total of 998 representative *O. rufipogon* accessions from fourteen regions of Guangdong and Hainan Provinces were used for this study. Of these accessions, 19 were from Dongguan, 48 were from Foshan, 60 were from Guangzhou, 38 were from Heyuan, 232 were from Huizhou, 128 were from Jiangmen, 74 were from Jieyang, 90 were from Maoming, 53 were from Qingyuan, 120 were from Shanwei, 15 were from Shenzhen, 22 were from Yangjiang, 52 were from Zhanjiang, and 47 were from Hainan (**Figure 1**). All materials were stored in the National Germplasm Guangzhou Wild Rice Nursery, China.

### Multiplex PCR, NGS and SNP calling

Genomic DNA was isolated with the cetyltrimethylammonium bromide (CTAB) method from young leaves of each *O. rufipogon* accession (Allen et al., 2006). A total amount of 100 ng genomic DNA per sample was used as input material for the DNA sample preparation. Sequencing libraries were generated using a MultipSeq^®^ Custom Panel (iGeneTech, Beijing, China) following the manufacturer’s recommendations, and index codes were added to each sample.

A total of 456 pairs of sample-specific multiplex PCR primers were designed and PCR amplification was performed at iGeneTech Bioscience Co., Ltd. The first round of multiplex PCR was performed as follows: 95̊ initial denaturation for 3 min 30 s, then 98̊ denaturation for 20 s, 60̊ primer annealing and extension for 2 min for 20 cycles, and finally extension for 5 min at 72̊. Products were purified using AMPure XP beads. The corresponding adapter oligonucleotides were joined by the second round of PCR, with the following procedure: 95°C initial denaturation for 3 min 30 s, 98°C denaturation for 20 s, 58°C primer annealing for 1 min, and 72°C extension for 30 s, with 10 cycles and finally extension for 5 min at 72°C. Products were purified using AMPure XP beads. Qubit^®^ 3.0 was used to determine the concentration of the library. An Agilent 2100 Bioanalyzer system was used to determine the length of the library fragments. Qualified libraries were sequenced on an Illumina platform.

Raw reads were filtered to remove low-quality reads by using FastQC. After cutting primer sequences, clean reads were mapped to the reference genome of *Oryza sativa* MSU V7.0 (Kawahara et al., 2013) by using Bwa. The detailed reads information was listed in **Supplementary Table S1**. Then, duplications were removed, and SNPs and InDels were called and annotated by using GATK and SAMtools.

### Phylogenetic and population genomic analyses

SNPs with a minor allele frequency (MAF) ≥ 1% and integrity ≥ 50% were used for phylogenetic and population structure analyses. The genetic diversity parameters of *O. rufipogon* were calculated using PowerMarker V3.25 (Liu and Muse, 2005). Principal component analysis (PCA) of the selected SNPs was performed with Cluster software (de Hoon et al., 2004). The ADMIXTURE program was used to assess population structure based on the maximum likelihood method with 10,000 iterations, and the number of clusters (*K*) was set from 1 to 10 (Alexander et al., 2009).

### Core collection construction and evaluation of *O. rufipogon*

A subset of *O. rufipogon* accessions were selected based on the strategy of population priority and gradual clustering. First, according to the geographical distribution of *O. rufipogon*, the accessions from the same population were divided into a group, and only one *O. rufipogon* accession in the group was directly selected into the core germplasm. Then, the genetic diversity and cluster analysis of *O. rufipogon* germplasms in each group were carried out with the SNP data, and the core germplasm was screened according to different sampling ratios to capture most of the allelic diversity in the germplasm using Core Hunter II software (Thachuk et al., 2009). Finally, the core collection was evaluated for genetic diversity and gene coverage and PCA was performed. Additionally, a phylogenetic tree was constructed using MEGA X software with the neighbour-joining (NJ) method (Kumar et al., 2018).

## Results

### Genotyping by multiplex PCR

**Figure 2:**
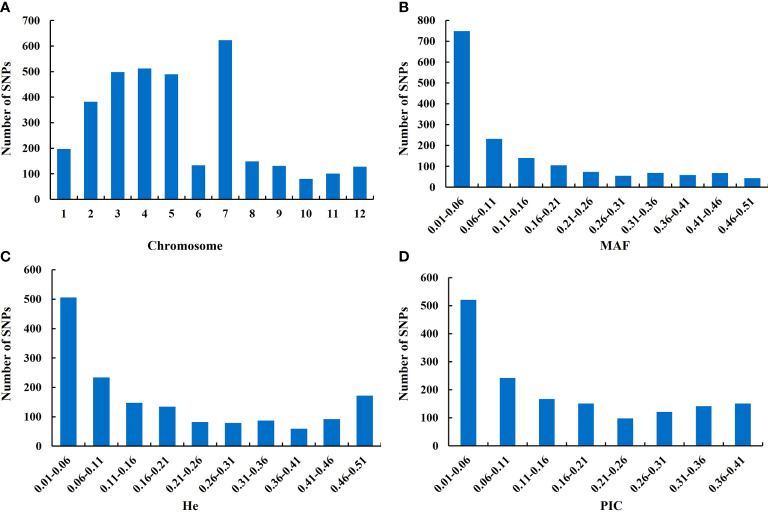
Characteristic statistics of SNPs. **(A)** SNP distribution on the rice scaffolds; **(B)** minor allele frequency (MAF); **(C)** expected heterozygosity (He); **(D)** polymorphism information content (PIC). The *Y*-axis represents the number of SNPs.

A total of 998 *O. rufipogon* accessions were sequenced and genotyped by multiplex PCR and NGS. The SNPs were further filtered under the condition of MAF ≥ 1% and integrity ≥ 50%, and 1,592 SNPs were generated for genetic diversity analysis. These SNPs were mapped onto 12 chromosomes. The highest and lowest SNPs mapped per chromosome were 623 and 80 on chromosome 7 and chromosome 10, respectively (**Figure 2A**). To explain the total variability of each marker, the MAF, the expected heterozygosity (He), and polymorphic information content (PIC) were used. The average MAF value was 0.128, with a minimum of 0.01 and a maximum of 0.499 (**Figure 2B**). The He was in the range of 0.02-0.50 with an average of 0.187 (**Figure 2C**). The PIC values ranged from 0.02 to 0.375, with an average of 0.157 (**Figure 2D**).

### Population structure and genetic relationships of *O. rufipogon*

**Figure 3:**
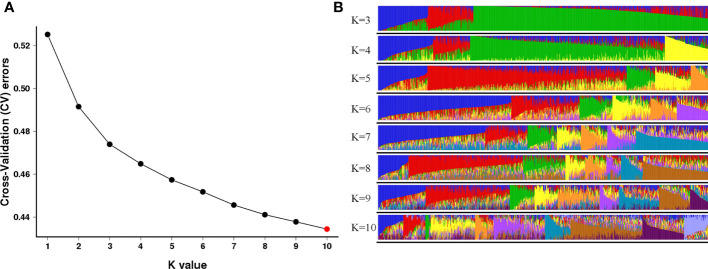
Population structure analyses of *O. rufipogon* accessions using ADMIXTURE. **(A)** Cross-validation plot for the number of population (*K*) values; **(B)** Model-based clustering with *K* values from 3 to 10. The colours represent different populations of the 998 wild rice accessions.

Population structure and genetic relationships were further examined to gain insight into the genetic diversity of *O. rufipogon* germplasm. The cross-validation errors (CVs) were examined under the models with *K* = 1-10 using admixture software. As suggested, a good K value will exhibit the lowest CV compared to other *K* values (Alexander et al., 2009). Here, the values of the CV continuously decreased with *K* from 1 to 9, and the minimum value was found when *K* = 10 (**Figure 3A**), which indicated that the 998 *O. rufipogon* accessions were classified into ten groups. The first and seventh groups both contained 75 accessions from 11 regions and six regions, respectively. The second group contained 65 accessions from seven regions. The 16, 131 and 55 wild rice accessions were divided into groups three, four and five, which came from four, fourteen and five regions. Groups six and eight contained 152 and 212 accessions, respectively, from 13 regions. The ninth group contained 124 *O. rufipogon* accessions from 12 regions, and the remaining 93 accessions were assigned to group 10 from 12 regions (**Figure 3B**). According to the geographical information of *O. rufipogon*, it was found that the germplasm resources of the ten different groups were geographically interspersed. The genetic relationship of some materials from different zones was close, indicating that there was mutual penetration between *O. rufipogon* accessions of different populations.

### Cluster analysis and genetic diversity comparison of *O. rufipogon* in different regions

*O. rufipogon* accessions of Guangdong Province were distributed in 13 different regions, and all collections in Hainan were divided into one region separately, of which genetic diversity comparisons were conducted (**Table 1**). All the SNP markers were found to be polymorphic, and an average of 758 polymorphic markers in each region were identified. The MAF varied between 0.2139 and 0.2422 with a mean value of 0.2275. The observed heterozygosity (Ho) and expected heterozygosity (He) of *O. rufipogon* accessions in Hainan were higher than that of those from the other thirteen regions. The Shannon–Wiener index (I), representing the genetic diversity of populations, had a value of 0.5022 in the Hainan subgroup, while the I in the other subgroups was lower than 0.5. The range of the PIC was from 0.248 to 0.2674, with an average of 0.2576. Nei’s diversity index ranged from 0.3047 to 0.3347, with an average of 0.32.

**Table 1:** Genetic diversity parameters of *O. rufipogon* in different collection city.

Group	Average_MAF	Np	Na	Ne	Nei	I	PIC	Obs_Het	Exp_Het
Dongguan	0.2215	778	2	1.5069	0.3186	0.4771	0.2529	0.2823	0.3101
Foshan	0.2202	770	2	1.5012	0.3111	0.4753	0.2518	0.3172	0.3079
Guangzhou	0.2139	799	2	1.4872	0.3047	0.4688	0.248	0.2987	0.3021
Heyuan	0.2341	760	2	1.5351	0.3301	0.4978	0.265	0.3376	0.3258
Huizhou	0.2218	777	2	1.5055	0.31	0.4767	0.2525	0.3114	0.3093
Jiangmen	0.2217	788	2	1.5039	0.3094	0.4752	0.2516	0.3225	0.3081
Jieyang	0.2333	737	2	1.5294	0.3236	0.492	0.2615	0.3199	0.3214
Maoming	0.2347	738	2	1.5339	0.3235	0.4915	0.261	0.3396	0.3217
Qingyuan	0.2199	791	2	1.503	0.3139	0.4802	0.2547	0.2938	0.311
Shanwei	0.2241	771	2	1.5104	0.3128	0.4795	0.2541	0.3026	0.3115
Shenzhen	0.2289	726	2	1.5237	0.3313	0.491	0.261	0.3277	0.3202
Yangjiang	0.2347	698	2	1.5319	0.3284	0.4907	0.2605	0.3371	0.3208
Zhanjiang	0.2345	731	2	1.5383	0.3284	0.496	0.2638	0.3045	0.3253
Hainan	0.2422	752	2	1.5547	0.3347	0.5022	0.2674	0.3465	0.3311
Mean	0.2275	758	2.0000	1.5189	0.3200	0.4853	0.2576	0.3172	0.3162

Number of poly markers, Na, Observed allele number; Ne, Expected allele number; I, Shannon–Wiener index; Ho, Observed heterozygosity; He, Expected heterozygosity; Nei, Nei diversity index; PIC, Polymorphism information content.

**Figure 4:**
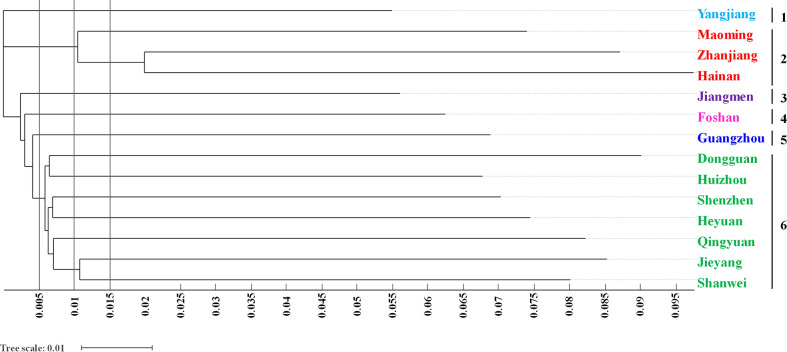
Dendrogram of *O. rufipogon* accessions in Guangzhou and Hainan using the NJ method. Different colours depict the population generated by the structure analysis.

With phylogenetic analysis, a neighbour-joining tree based on Nei’s genetic distance was also clearly constructed. The 998 *O. rufipogon* accessions of 14 regions were grouped into six main clusters when the tree scale was 0.005 (**Figure 4**). Cluster 1 contained accessions from Yangjiang, and the accessions from Maoming, Zhanjiang and Hainan tended to be grouped together in cluster 2. Cluster 3, 4 and 5 were characterized as independent groups of *O. rufipogon* containing accessions from Jiangmen, Foshan, and Guangzhou, respectively. All the accessions from seven regions (Dongguan, Huizhou, Shenzhen, Heyuan, Qingyuan, Jieyang and Shanwei) were restricted to the major cluster 6. When the tree scale was 0.01, all *O. rufipogon* accessions were clustered into 11 groups. The accessions from Dongguan and Huizhou were grouped together, and the accessions from Qingyuan was grouped separately. Moreover, another cluster containing accessions from Jieyang and Shanwei were characterized.

### *O. rufipogon* core collection establishment

**Figure 5:**
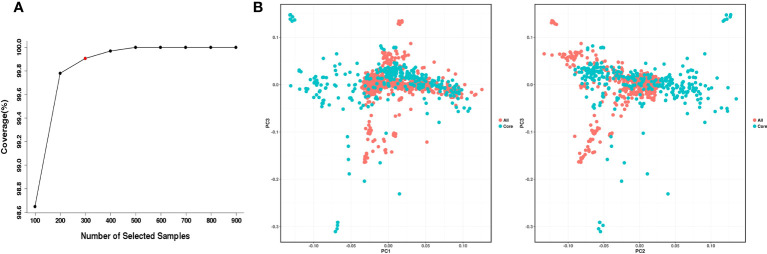
Development and evaluation of the *O. rufipogon* core collection. **(A)** Coverage of allelic diversity versus number of selected accessions. The red circle indicates the minimal number of samples (299) covering 99.906% allelic diversity. **(B)** PCA of *O. rufipogon* accessions. Red dots represent the accessions in the core collection; green dots represent the accessions not in the core collection.

Construction of a core collection provides a subset of representative accessions that captures most of the allelic diversity in the entire collection and can be used for genome-wide association studies (GWAS), gene cloning and marker development. According to the geographical distribution and genotype information of 998 *O. rufipogon* accessions, the core germplasm was developed using Core Hunter II software with a modified Rogers distance of 0.7 and Shannon diversity index of 0.3. Our analysis indicated that the 499 top-ranked accessions could capture 100% of the allelic diversity of the entire *O. rufipogon* resource collection (**Figure 5A**). A core set of 299 accessions was selected from 998 *O. rufipogon* accessions, capturing 99.906% of the entire allelic diversity. The top two groups with the highest proportions in the 299-accession core collection were from Huizhou (44; 14.7%) and Shanwei (37; 12.4%), while the proportion of core germplasm from Shenzhen and Yangjiang was relatively low, accounting for only 0.67% and 2.34% of the total core germplasm, respectively. The detailed information is listed in **Table 2**.

**Table 2:** The origins of core germplasms of *O. rufipogon* in Guangdong.

Number	Collection region	Number of populations	Number of core germplasms
1	Dongguan	7	10
2	Foshan	6	9
3	Guangzhou	3	16
4	Heyuan	5	15
5	Huizhou	24	44
6	Jiangmen	7	25
7	Jieyang	8	20
8	Maoming	16	36
9	Qingyuan	9	18
10	Shanwei	17	37
11	Shenzhen	1	2
12	Yangjiang	6	7
13	Zhanjiang	14	28
14	Hainai	11	32
	Total:	134	299

### Evaluation of the core collection

To further verify the accuracy of the core accessions, the genetic diversity of 299 *O. rufipogon* resources was determined (**Table 3**). The coverage of polymorphic markers in the core collections was 95.35% of those detected in the whole germplasm. The Ne retained 1.336 and 1.291 in core collections and the whole germplasm. The mean Nei’s diversity index and PIC value of this core collection were 0.215 and 0.179, accounting for 114.97% and 114.01% of the total accessions, respectively, supporting the representativeness of the core collections. In addition, the Shannon–Wiener index, Ho and He between the representative set and the total collection were 1.12, 1.06 and 1.14, respectively, indicating a high degree of similarity.

**Table 3:** Genetic diversity parameters between core germplasm and original germplasm.

Type	Number	Average MAF	Np	Na	Ne	Nei	I	PIC	Ho	He
Total sample	998	0.129	1592	2	1.291	0.187	0.308	0.157	0.175	0.187
core germplasm	299	0.148	1518	2	1.336	0.215	0.347	0.179	0.186	0.214
Percentage of core germplasm (%)	29.96	114.73	95.35	100.00	103.49	114.97	112.66	114.01	106.29	114.44

PCA of the accessions in the *O. rufipogon* core collection exhibited a pattern nearly identical to that of the accessions in the entire collection (**Figure 5B**), further supporting the broad representation of the wild rice accessions in the core accessions from Guangdong and Hainan Provinces.

**Figure 6:**
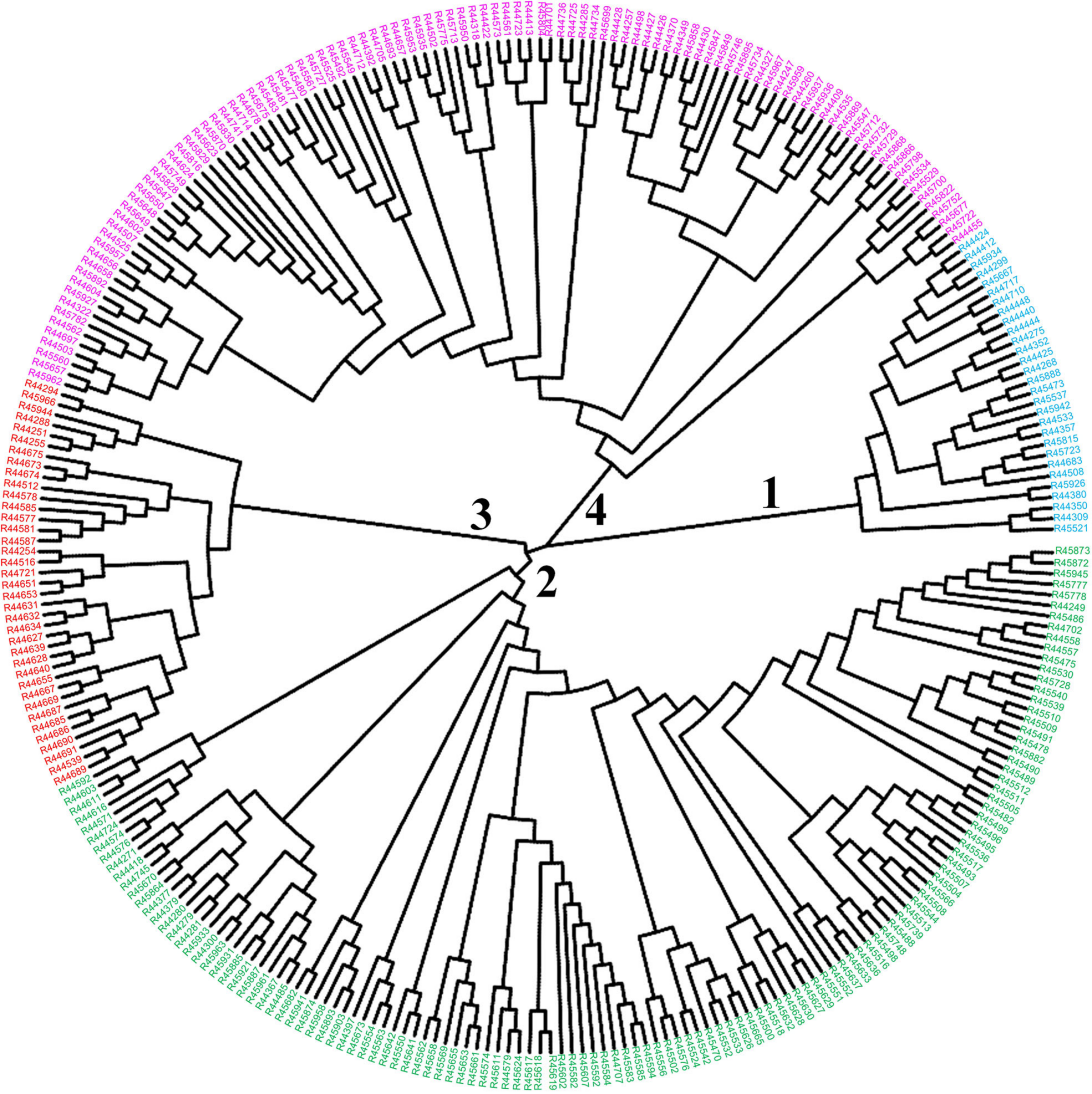
Neighbour-joining tree of the core collection of *O. rufipogon*. Colours represent different subpopulations of the core collection; blue colour = subpopulation 1, green = subpopulation 2, red = subpopulation 3, purple = subpopulation 4.

To infer phylogenetic relationships among the 299 *O. rufipogon* accessions, a neighbour-joining phylogenetic tree was constructed with the identified SNPs. Four major clades were identified in the core collections, with the red cluster tending to contain accessions from five regions in Guangdong Province. Similarly, the blue cluster mainly comprised accessions from ten regions in Guangdong and Hainan Provinces. The remaining two clades contained accessions from 13 and 14 regions, respectively (**Figure 6**). There was some overlap in the accessions from the 14 regions among the subgroups.

## Discussion

### Evaluation of the genetic diversity of *O. rufipogon*

*O. rufipogon* is an important genetic resource for rice breeding. Guangdong and Hainan are important distribution areas of *O. rufipogon* in China. Due to increased industrialization and the expansion of economic production activities, the original habitat of wild rice has been destroyed, and the distribution area has been greatly reduced. Thus, there is an urgent need to study the genetic diversity of *O. rufipogon* resources. Currently, array-based SNP detection is one of the major high-throughput marker detection platforms and can be used to genotype multiple samples and study the genetic characteristics of plants within a short period (Zhu et al., 2019). In this study, a total of 998 *O. rufipogon* accessions from 14 regions of Guangdong and Hainan Provinces were sequenced and genotyped by multiplex PCR and NGS, and 1,592 SNPs were generated for genetic diversity analysis. The range of the Shannon–Wiener index (I) was from 0.056 to 0.693, with an average of 0.308. Nei’s diversity index ranged from 0.02 to 0.5, with an average of 0.187. The results revealed that *O. rufipogon* populations of Guangdong and Hainan Provinces had rich genetic diversity and that variations were widely distributed in the populations. However, if the number of SNPs would be further increased, the more perfect genetic relationships would be obtained. There were also differences in the genetic diversity of *O. rufipogon* resources in 14 regions, which suggested that the geographical environment might be responsible for the genetic background differences in *O. rufipogon* in different regions. It was consistent with previous research result (Zhang et al., 2021). We detected high levels of genetic diversity in *O. rufipogon* populations from Guangdong Province. For example, the Shenzhen and Heyuan populations had a higher Nei’s diversity index and Shannon–Wiener index, PIC, and He, respectively, while the Guangzhou population had the lowest level of diversity. Furthermore, *O. rufipogon* accessions from Hainan Province had the highest level of diversity compared to those from the other 13 regions, possibly because of the extensive habitat distribution of populations in Hainan. At the same time, due to the small amount of *O. rufipogon* accessions collected in each subregion of Hainan, the diversity comparison of populations within Hainan Province was limited.

*O. rufipogon* accessions were clustered according to the genetic similarity of each region. Populations geographically close to each other were often clustered into one group and were more closely related. As depicted in **Figure 4**, the accessions from Yangjiang tended to be grouped in cluster 1, while all the accessions from Jieyang and Shanwei were restricted to cluster 6. The results suggested that the genetic relationship among the *O. rufipogon* accessions from the distribution sites was closely related to geographical location. In addition, 998 *O. rufipogon* accessions had clear group divisions with K values from 2 to 10, and there was mutual infiltration between the germplasm resources of different groups in terms of geographical origin.

In Guangdong and Hainan Provinces, *O. rufipogon* accessions are widely distributed and difficult to protect. The results of genetic diversity analysis provide a scientific basis for the protection of these *O. rufipogon* resources. Conservation efforts should be made based on the geographical distribution characteristics of the genetic diversity of *O. rufipogon*. Accordingly, *ex situ* conservation in the Heyuan, Zhanjiang, Huizhou, Jiangmen and Hainan areas with high genetic diversity and large numbers of germplasms is a good strategy for preserving the genetic diversity of these important wild rice varieties. At the same time, the collected wild rice accessions should contain the accessions of each population during *in situ* conservation and core collection construction to maximize the genetic diversity of wild rice resources.

### A core germplasm to represent the maximum genetic diversity of the total *O. rufipogon* accessions

The construction of core collections could improve the efficiency of the utilization of germplasm resources. For instance, core collections can be used for the development of molecular markers, GWAS of important traits, rice domestication and diversity study (Agrama et al., 2009; Song et al., 2018; Wang et al., 2018; Zhang et al., 2019). Although there are multiple ways to construct core germplasms, the evaluation criteria established for core germplasms are largely similar, that is, that the diversity be representative of the original germplasm. A mini core collection consisting of 189 varieties of *Oryza sativa* was developed in China (Zhang et al., 2011), and a total of 701 accessions were developed that accounted for approximately 10% of accessions from the total North-Eastern region of India with the unweighted pair group method of arithmetic average (UPGMA) (Roy et al., 2014). Moreover, 130 wild rice accessions from China were selected as a core collection that retained over 90% of the alleles (Liu et al., 2016).

In this study, we used the NJ method and genetic distance in combination with population geographic distributions to develop the wild rice core collection. A core set of 299 accessions was established. Genetic diversity tests, PCA, and phylogenetic analysis were performed on the core and entire collection to ensure the genetic diversity of the core collection. The results showed that 29% of the sampling percentage selected from the entire germplasm retained 99.906% of the entire allelic diversity. In addition, the core collection contained accessions from all populations in Guangdong and Hainan Province. Briefly, the core germplasm maintained a high level of genetic diversity and was representative of the entire population.

In conclusion, we assembled a core collection of *O. rufipogon* with abundant genetic diversity from different ecological regions of Guangdong and Hainan Provinces, China, and our strategy was successful in selecting representative accessions from the entire population based on genotypic data. Furthermore, it is critical that *ex situ* and *in situ* conservation of the *O. rufipogon* germplasm be undertaken to avoid inter- and intrapopulation introgression. Finally, the core collections constructed in this study are valuable wild rice resources, laying a solid material foundation to promote the identification and utilization of superior genes of *O. rufipogon* resources in Guangdong and Hainan Provinces in the future.

## Data availability statement

The original contributions presented in the study are included in the article/**Supplementary Material**. Further inquiries can be directed to the corresponding authors.

## Author contributions

LC conceived the research. ZJ and LQ performed the research. ZJ wrote the paper. ZJ, PD, FZ, YH, JL, LS, SB CW and MX participated in the preparation of the reagents in this study. All authors contributed to the article and approved the submitted version.

## Funding

This work was supported by grants from Guangdong Provincial Key Research and Development Project Program (2020B0202090003), the Special Fund for Science and Technology Innovation Strategy (Construction of High-Level Academy of Agricultural Sciences) (201926), the Subproject of National Key Research and Development Program (2021YFD1200101-05) and Guangdong Key Laboratory of New Technology in Rice Breeding (2020B1212060047).

## Conflict of interest

The authors declare that the research was conducted in the absence of any commercial or financial relationships that could be construed as a potential conflict of interest.

## Publisher's note

All claims expressed in this article are solely those of the authors and do not necessarily represent those of their affiliated organizations, or those of the publisher, the editors and the reviewers. Any product that may be evaluated in this article, or claim that may be made by its manufacturer, is not guaranteed or endorsed by the publisher.
